# Integrating epidemiology with genomic tracing to uncover concealed transmission networks in a brucellosis outbreak, Shandong, China

**DOI:** 10.3389/fcimb.2026.1794629

**Published:** 2026-04-21

**Authors:** Chanjuan Huangfu, Min Wang, Junjie Fan, Xiujun Ma, Haizong Zhang, Jing Yuan, Kuo Han, Ti Liu, ShuJun Ding, Zengqiang Kou, Yan Li

**Affiliations:** 1 School of Public Health and Health Management, Shandong First Medical University, Jinan, China; 2 Clinical Laboratory, Children's Hospital Affiliated to Shandong University, Jinan Children's Hospital, Jinan, China; 3 Department of Infectious Disease Prevention and Control, Weifang Center for Disease Control and Prevention, Weifang, China; 4 Shandong Provincial Key Laboratory of Intelligent Monitoring, Early Warning, Prevention and Control for Infectious Diseases, Shandong Center for Disease Control and Prevention, Jinan, China; 5 Department of Infectious Disease Prevention and Control, Linqu County Center for Disease Control and Prevention, Weifang, China

**Keywords:** *Brucella melitensis*, brucellosis, molecular epidemiology, molecular typing, outbreaks, whole-genome sequencing

## Abstract

**Background:**

This study integrated epidemiological investigation with whole-genome sequencing to elucidate the transmission chain and pathogen characteristics of a 2023 brucellosis outbreak in Shandong Province, providing evidence for targeted prevention and control.

**Methods:**

Transmission chains were reconstructed through field epidemiological investigations, active case finding, and retrospective data review. Isolated strains were cultured and identified, followed by molecular tracing using multilocus variable-number tandem repeat analysis (MLVA) and core-genome single-nucleotide polymorphism (cgSNP) genotyping. Epidemiological and molecular data were integrated to assess transmission links.

**Results:**

A total of 26 related cases were identified in this outbreak. Epidemiological investigation revealed that the outbreak originated from a flock of infected lambs introduced by an index case in 2022, with subsequent spread through local livestock trading networks affecting multiple villages. Ten *Brucella* strains were isolated, including nine of *Brucella melitensis* biovar 3 (*B. melitensis* bv. 3) and one of *B. melitensis* bv. 1. cgSNP analysis showed that the nine bv. 3 strains formed a single clonal cluster (0 SNP difference) and grouped with historical Shandong strains, indicating sustained local transmission. The bv. 1 strain represented a separate infection event, genetically distinct from the main outbreak cluster. Integrated analysis confirmed a complete molecular-epidemiological concordance for 38.5% (10/26) of cases; the remaining 61.5% could not be definitively linked due to missing epidemiological data or lack of isolate recovery. Phylogenetic analysis further indicated co-circulation of multiple lineages in the region.

**Conclusion:**

This outbreak was primarily driven by the trade of locally infected sheep, facilitating regional spread. High-resolution molecular typing effectively complemented traditional epidemiology by uncovering concealed transmission chains and revealing the co-circulation of multiple lineages. These findings underscore the value of integrated molecular surveillance systems in key endemic regions to support human brucellosis control.

## 1 Introduction

Brucellosis is a widespread zoonotic disease caused by facultative intracellular bacteria of the genus *Brucella* (Percin, 2013; Głowacka et al., 2018). Humans are primarily infected through direct contact with infected animals or by consuming contaminated animal products (Holzhauer and Wennink, 2023; Almashhadany et al., 2026). Approximately 500,000 new cases are reported globally each year, affecting over 170 countries and imposing a significant public health burden (Franc et al., 2018). In China, the incidence of brucellosis remains persistently high, with a concerning geographical expansion from traditional northern endemic regions to southern coastal and southwestern provinces (Liu et al., 2024, Liu et al., 2025). *B. melitensis* is the predominant species causing human brucellosis worldwide; its biovar 1 (bv. 1) and biovar 3 (bv. 3) are particularly common and are frequently associated with outbreaks and recurrent epidemics (An et al., 2024). Shandong Province is a key endemic area, with Weifang City being particularly severely affected. Surveillance data show that the incidence rate in Weifang rebounded sharply from 2.81 per 100,000 in 2021 to 4.17 per 100,000 in 2023, marking an increase of 48.4%. This high-risk setting was closely associated with the largest brucellosis cluster outbreak in the province that year, which was first identified in Weifang in 2023. Brucellosis is a classic neglected zoonosis, imposing a significant economic burden on affected households, particularly in rural and pastoral communities. Studies from endemic areas, including Kazakhstan and China’s Xinjiang, highlight substantial out-of-pocket costs for patients, which can lead to medical impoverishment among vulnerable groups such as farmers and herders (Charypkhan et al., 2019; He et al., 2024). Diagnostic delays further exacerbate this burden by increasing the risk of costly complications.

Field epidemiological investigations are crucial for outbreak management, enabling rapid identification of infection sources, transmission chains, and high-risk populations (Yuan et al., 2018). However, their effectiveness depends on the completeness and timeliness of data. Moreover, they are susceptible to reporting delays, information gaps, and recall bias. These limitations can lead to inaccurate conclusions and delayed interventions (Cowling et al., 2008; Gaulin et al., 2014). To overcome these limitations, high-resolution molecular tools have been increasingly adopted. Multilocus variable-number tandem repeat analysis (MLVA), with its standardized 16-locus panel, enables rapid preliminary screening and direct comparison with publicly available databases for global strain comparison (Dadar and Alamian, 2025). Advances in whole-genome sequencing (WGS) technology have substantially addressed these limitations (Ksibi et al., 2025). High-resolution molecular typing methods, such as core-genome single-nucleotide polymorphism (cgSNP) analysis, provide powerful tools for elucidating intricate phylogenetic relationships and transmission dynamics among pathogens and have been increasingly applied in outbreak investigations (Wang et al., 2025). In this study, we investigated a major brucellosis outbreak in Weifang City, Shandong Province, in 2023 by integrating detailed field epidemiology with cgSNP analysis for genomic tracing. Our objectives were to precisely reconstruct the outbreak’s transmission network, assess the genetic relationship between the outbreak strain and local as well as nationally circulating lineages, and establish a robust genomic evidence base for understanding regional epidemiological patterns of *Brucella* and for informing targeted surveillance and control strategies.

## 2 Methods

### 2.1 Epidemiological investigation

#### 2.1.1 Data sources and case definitions

Human brucellosis incidence data were sourced from the Infectious Disease Surveillance System of the Chinese Center for Disease Control and Prevention. Detailed outbreak information was collected through on-site interviews using standardized epidemiological questionnaires. The original questionnaire was developed in Chinese by the Chinese Center for Disease Control and Prevention. An English translation is provided in **Supplementary Table 1**. Case confirmation adhered to China’s Diagnostic Criteria for Brucellosis (WS 269-2019) (National Health Commission of China, 2019). A confirmed case was defined as an individual with an epidemiologically plausible exposure history, consistent clinical symptoms, and serological confirmation by both a positive rose Bengal plate test (RBPT) and a standard tube agglutination test (SAT) titer of ≥1:100 (++).

Cases were defined as outbreak-associated if they had an epidemiological link to the index case’s infected flock (documented sheep purchase or contact) or genetic relatedness to the outbreak strain (≤7 SNP differences). Based on the availability of bacterial isolates and the concordance between epidemiological and molecular evidence, cases were classified into distinct groups for integrated analysis.

Diagnostic delay was defined as the time interval (in days) between the date of illness onset and the date of diagnosis, both of which are recorded as standard fields in the Infectious Disease Reporting Card of the China Information System for Disease Control and Prevention. Descriptive statistics were used to summarize the demographic and clinical characteristics of the outbreak-associated cases. Categorical variables were presented as frequencies and percentages, and continuous variables (age, diagnostic delay) were presented as median with range. All data analyses were conducted using Microsoft Excel 2021.

#### 2.1.2 Field investigation and case search

To comprehensively elucidate the transmission chain, we implemented active case finding using multiple strategies: (1) Transmission chain tracing: Starting from the index case’s sheep sales records, we conducted backward tracing to identify all buyers, their household members, and close contacts. (2) Key population screening: We performed symptom screening and serological testing (RBPT and SAT) on all livestock farmers and their household members in villages linked to confirmed cases. (3) Medical record review: Outpatient, emergency, and inpatient records from all healthcare facilities in and near the outbreak area during the epidemic period were reviewed to identify missed or misdiagnosed cases. (4) Household cluster analysis: We analyzed family relationships and the timing of symptom onset for all cases, focusing on households with multiple cases occurring within a short interval (typically 3 to 6 months) to identify transmission within households.

All field investigations were conducted by trained public health staff. After obtaining verbal informed consent, suspected and confirmed cases were interviewed face-to-face using a structured questionnaire to collect demographic details, clinical symptoms, date of onset, and potential exposure history.

### 2.2 Laboratory strain isolation, culture and identification

#### 2.2.1 Isolation and culture

Venous blood (5-10 mL) was collected from patients and inoculated into blood culture bottles for enrichment. A 100 μL aliquot of the enriched broth was then streaked onto Columbia blood agar plates and incubated at 37 °C with 5% CO_2_ for 48-72 h. Colonies exhibiting typical morphology were selected and subcultured. A single colony from a 48h pure culture was suspended in 200 μL of sterile saline. The bacterial suspension was heat-inactivated at 80 °C for 90 min using a heating block. Genomic DNA was extracted with a commercial bacterial DNA extraction kit (TianGen, Beijing, China) and stored at -20 °C for further molecular typing and whole-genome sequencing. All work with live *Brucella* was conducted in a Biosafety Level 3 (BSL-3) laboratory in compliance with national biosafety regulations for pathogenic microorganisms.

#### 2.2.2 Strain identification and biotyping

Preliminary strain identification and biotyping were performed according to the national standard (WS269-2019) (National Health Commission of China, 2019). Molecular identification was carried out using BCSP31-PCR for genus-level confirmation (Fekete et al., 1990) and AMOS-PCR for species-level discrimination (Bricker and Halling, 1994).

### 2.3 Whole-genome sequencing and molecular typing

#### 2.3.1 Whole-genome sequencing and assembly

Genomic DNA was submitted to Novogene (Tianjin, China) for paired-end sequencing (2 × 150 bp) on an Illumina NovaSeq X Plus platform. Raw reads were quality-assessed using FastQC (v0.11.9) and trimmed with fastp (v0.21.0) to generate clean data. *De novo* assembly was performed using SPAdes (v3.15.5). All assembled genomes met high quality standards based on standard metrics (e.g., N50, GC content, scaffold count) and were used for subsequent analyses.

#### 2.3.2 MLVA typing

In silico MLVA typing was conducted based on the assembled whole-genome sequences using CLC Genomics Workbench 23.0.3 (QIAGEN, Germany). The copy numbers across 16 variable-number tandem repeat (VNTR) loci were determined by comparing the amplified fragment lengths with known repeat unit sizes. Reference data for MLVA-16 were obtained from the MLVAbank (https://microbesgenotyping.i2bc.paris-saclay.fr/, accessed 10 May 2025). The resulting typing data were analyzed using the unweighted pair group method with arithmetic mean (UPGMA) algorithm in BioNumerics 8.0 (Applied Maths, Belgium).

#### 2.3.3 cgSNP analysis

Genome annotation for all assembled strains was performed using Prokka (v1.14.6). The core gene set was then extracted with Roary (v3.12.0) to generate a multi-sequence alignment. CgSNP sites were identified from this alignment using snp-sites (v2.5.1), producing a SNP alignment file (core_SNPs. aln) for phylogenetic analysis. A maximum-likelihood (ML) phylogenetic tree was constructed from this SNP matrix using MEGA X software (Mega Limited, New Zealand) with 1000 bootstrap replicates to assess node support. Strains differing by ≤7 SNPs were considered potentially epidemiologically linked, a threshold proposed as a potential criterion for *Brucella* outbreak investigations (Janowicz et al., 2018).

To contextualize the outbreak strains, all publicly available *B. melitensis* whole-genome sequences were downloaded from the NCBI database as a background dataset. Preliminary analysis revealed that strains closely related to the outbreak cluster were exclusively of Chinese origin. Therefore, the final phylogenetic reconstruction and visualization focused on the 10 outbreak strains, relevant strains from the Shandong Provincial surveillance database, and selected representative strains from other Chinese provinces, to optimize clarity and highlight key genetic relationships.

### 2.4 Integration of molecular and epidemiological data

**Figure 1:**
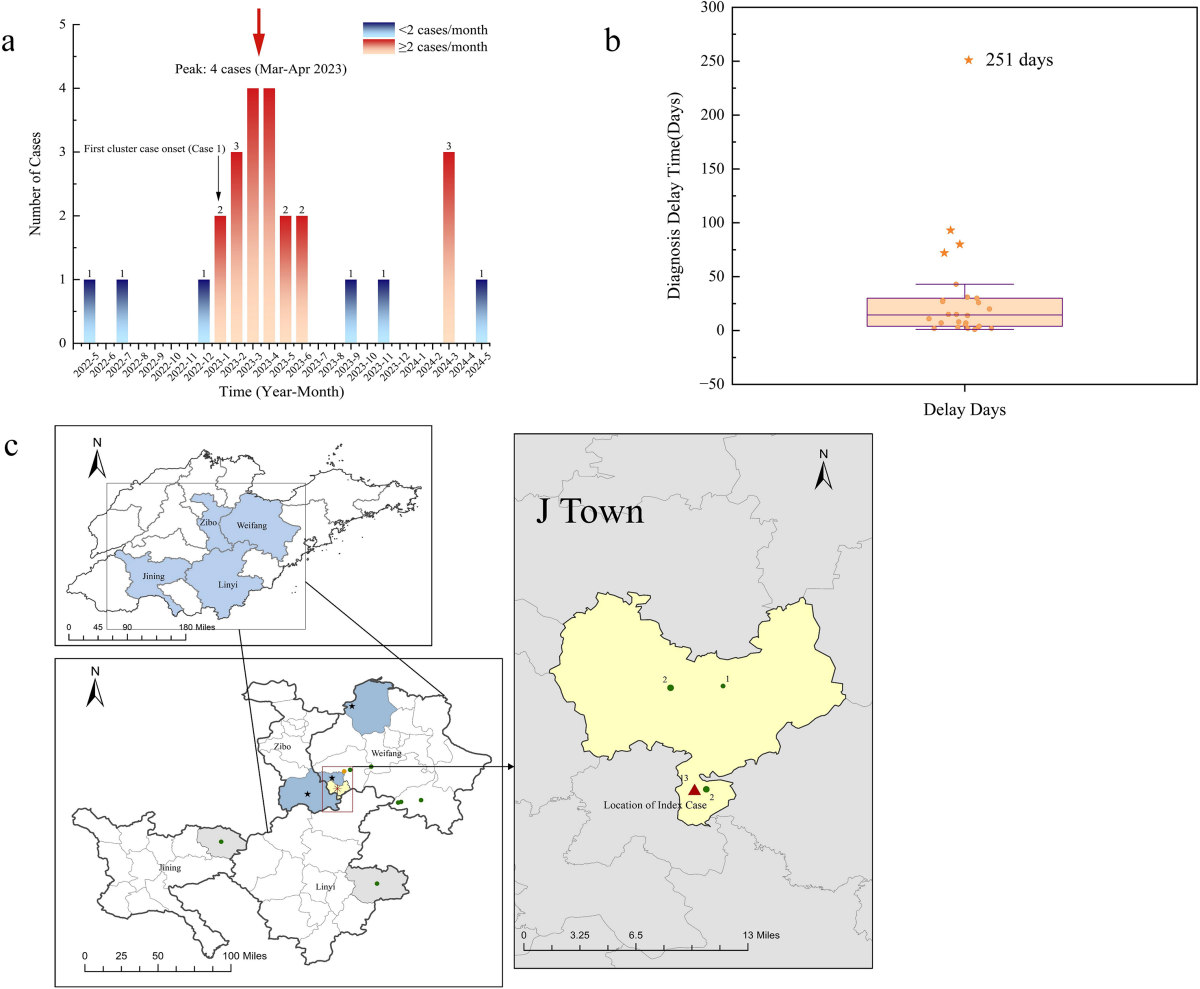
Spatiotemporal distribution characteristics of the brucellosis outbreak (May 2022 to May 2024). **(a)** Monthly distribution of case onset. **(b)** Box plot of diagnostic delay (in days). The outlier at 251 days is indicated. **(c)** Geographic distribution of cases. Top left: Provincial background map of Shandong Province, with blue areas indicating the four prefecture−level cities (Zibo, Weifang, Jining, Linyi) associated with this outbreak. Bottom left: Enlarged view of the four prefecture−level cities. Green dots indicate case locations (one case per dot). Orange dot: Case 18, exposed in L County (exact location unknown). Gray areas: two linked cases in Linyi and Jining, identified by cgSNP analysis (≤7 SNPs to the core outbreak strain). Black stars: source locations of sheep purchased by the index case (Y County, Zibo City; S and T Towns, L County). Right: Enlarged view of the core epidemic focus (J Town). Yellow area: J Town boundary. Green dots: case locations, with numbers indicating case counts. Red triangle: the combined geographic location of Villages A and B, where a total of 13 cases (including the index case) occurred and could not be differentiated at the village level on the map. Villages A, B, C, D, and F are all within J Town.

To identify potential transmission links, a transmission network was reconstructed through stepwise integration of epidemiological and molecular data. Individual-level epidemiological variables (animal exposure history, contact tracing records, residential location, and symptom onset date) were collated and standardized in Microsoft Excel 2021. Molecular relatedness was defined by cgSNP analysis (≤7 SNPs indicative of epidemiological linkage) and MLVA-16 genotyping. Epidemiological and molecular evidence were integrated on a case-by-case basis to reconstruct putative transmission chains. The resulting network was spatially visualized in ArcGIS 10.8 (ESRI, USA) to characterize transmission clusters, inter-village spread, and cross-regional pathways. The final transmission network is summarized in **Table 1** and **Figure 1**.

**Table 1:** Epidemiological and molecular characteristics of brucellosis cases associated with the outbreak in Weifang, Shandong Province.

Case number	Strain number	Age	Gender	Onset date	Diagnosis date	Epidemiological association	Home address	Strain isolation	MLVA-16 typing	SNP difference	Association group
Index case (Sun)	2022SD222	54	Male	2022/05/17	2022/06/14	Yes	Village A, L County	Yes	1-5-3-13-2-2-3-2-4-41-8-5-4-3-6-6	0	Group 1
Case 1	2023SD508	65	Male	2023/01/11	2023/02/23	Yes	Village A, L County	Yes	1-5-3-13-2-2-3-2-4-41-8-5-4-3-7-6	0	Group 1
Case 2	2023SD507	53	Male	2023/02/03	2023/02/18	Yes	Village A, L County	Yes	1-5-3-13-2-2-3-2-4-41-8-5-4-3-7-6	0	Group 1
Case 3	2023SD509	53	Female	2023/02/04	2023/02/24	Yes	Village A, L County	Yes	1-5-3-13-2-2-3-2-4-41-8-5-4-3-7-6	0	Group 1
Case 4	2023SD506	64	Female	2023/02/04	2023/02/06	Yes	Village A, L County	Yes	1-5-3-13-2-2-3-2-4-41-8-4-4-3-7-5	129	Group 2
Case 5		66	Male	2022/07/01	2023/03/09	Yes	Village A, L County	No	–	–	Group 3
Case 6		55	Male	2022/12/13	2023/03/03	Yes	Village A, L County	No	–	–	Group 3
Case 7		67	Male	2023/03/29	2023/04/05	Yes	Village A, L County	No	–	–	Group 3
Case 8	2023SD517	61	Female	2023/03/29	2023/04/05	Yes	Village A, L County	Yes	1-5-3-13-2-2-3-2-4-41-8-5-4-3-7-6	0	Group 1
Case 9		71	Female	2023/05/07	2023/05/10	Yes	Village A, L County	No	–	–	Group 3
Case 10	2023SD1037	74	Male	2023/06/07	2023/07/04	Yes	Village A, L County	Yes	1-5-3-13-2-2-3-2-4-41-8-5-4-3-7-6	0	Group 1
Case 11		64	Female	2023/03/02	2023/03/13	Yes	Village B, L County	No	–	–	Group 3
Case 12		66	Male	2023/04/05	2023/04/06	Yes	Village B, L County	No	–	–	Group 3
Case 13	2023SD519	60	Female	2023/03/25	2023/04/20	Yes	Village C, L County	Yes	1-5-3-13-2-2-3-2-4-41-8-5-4-3-7-6	0	Group 1
Case 14		59	Male	2023/01/26	2023/04/29	Yes	Village C, L County	No	–	–	Group 3
Case 15	2023SD521	53	Female	2023/04/22	2023/04/27	Yes	Village D, L County	Yes	1-5-3-13-2-2-3-2-4-41-8-5-4-3-7-6	0	Group 1
Case 16	2023SD524	49	Male	2023/05/01	2023/05/03	Yes	Village D, L County	Yes	1-5-3-13-2-2-3-2-4-41-8-5-4-3-7-6	0	Group 1
Case 17	2023SD765	68	Female	2023/04/09	2023/04/23	No	Z County	Yes	1-5-3-13-2-2-3-2-4-41-8-5-4-3-7-6	0	Group 4
Case 18	2023SD525	8	Female	2023/04/06	2023/05/06	Yes	Weihai (infected in L County)	Yes	1-5-3-13-2-2-3-2-4-41-8-5-4-3-7-6	0	Group 1
Case 19	2023SD441	68	Male	2023/09/17	2023/10/02	No	C County	Yes	1-5-3-13-2-2-3-2-4-41-8-5-4-3-7-6	0	Group 4
Case 20	2023SD1045	48	Male	2023/11/17	2023/11/20	No	Village E, L County	Yes	1-5-3-13-2-2-3-2-4-41-8-5-4-3-7-6	0	Group 4
Case 21	2024SD333	71	Female	2024/03/03	2024/04/03	No	Z County	Yes	1-5-3-13-2-2-3-2-4-41-8-5-4-3-7-6	1	Group 4
Case 22	2024SD332	70	Male	2024/03/26	2024/03/30	No	Z County	Yes	1-5-3-13-2-2-3-2-4-41-8-5-4-3-7-6	1	Group 4
Case 23	2024SD399	63	Male	2024/05/21	2024/05/23	No	Village F, L County	Yes	1-5-3-13-2-2-3-2-4-41-8-5-4-3-7-6	0	Group 4
Case 24	2023SD754	57	Female	2023/06/11	2023/06/19	No	Linyi City	Yes	1-5-3-13-2-2-3-2-4-41-8-6-4-3-6-7	4	Group 4
Case 25	2024SD462	57	Female	2024/03/12	2024/05/23	No	Jining City	Yes	1-5-3-13-2-2-3-2-4-41-8-5-4-3-5-6	6	Group 4

SNP differences are shown relative to the consensus cgSNP genotype of the major outbreak clade (the nine identical *B. melitensis* bv. 3 strains); ≤7 SNPs indicate potential epidemiological linkage.

## 3 Results

### 3.1 Outbreak overview and epidemiological investigation

In February 2023, a brucellosis outbreak was identified in Village A of L County, Weifang City, Shandong Province. Hospitals initially reported four confirmed cases, including a married couple (Cases 2 and 3). Epidemiological investigation revealed a common exposure: all cases had purchased lambs in June 2022 from the same livestock farmer in Village A. This individual was subsequently confirmed as the index case (Mr. Sun). The index case had acquired over 30 lambs between January and April 2022 from Y County (Zibo City) and from S Town and T Town within L County. He developed symptoms in May 2022 and was diagnosed in June. Following his diagnosis, his flock was sold to farmers in Village A and in Villages B, C, and D within J Town.

To contain the outbreak, the L County Center for Disease Control and Prevention (CDC) implemented active surveillance from February to July 2023, including: (1) tracing all lamb purchasers and their close contacts based on sales records; (2) reviewing fever case records from health centers in the affected villages (June 2022 to July 2023); and (3) conducting serological testing (RBPT and SAT) on all livestock farmers and their household members (n=62) in Village A in March and July 2023.

**Figure 2:**
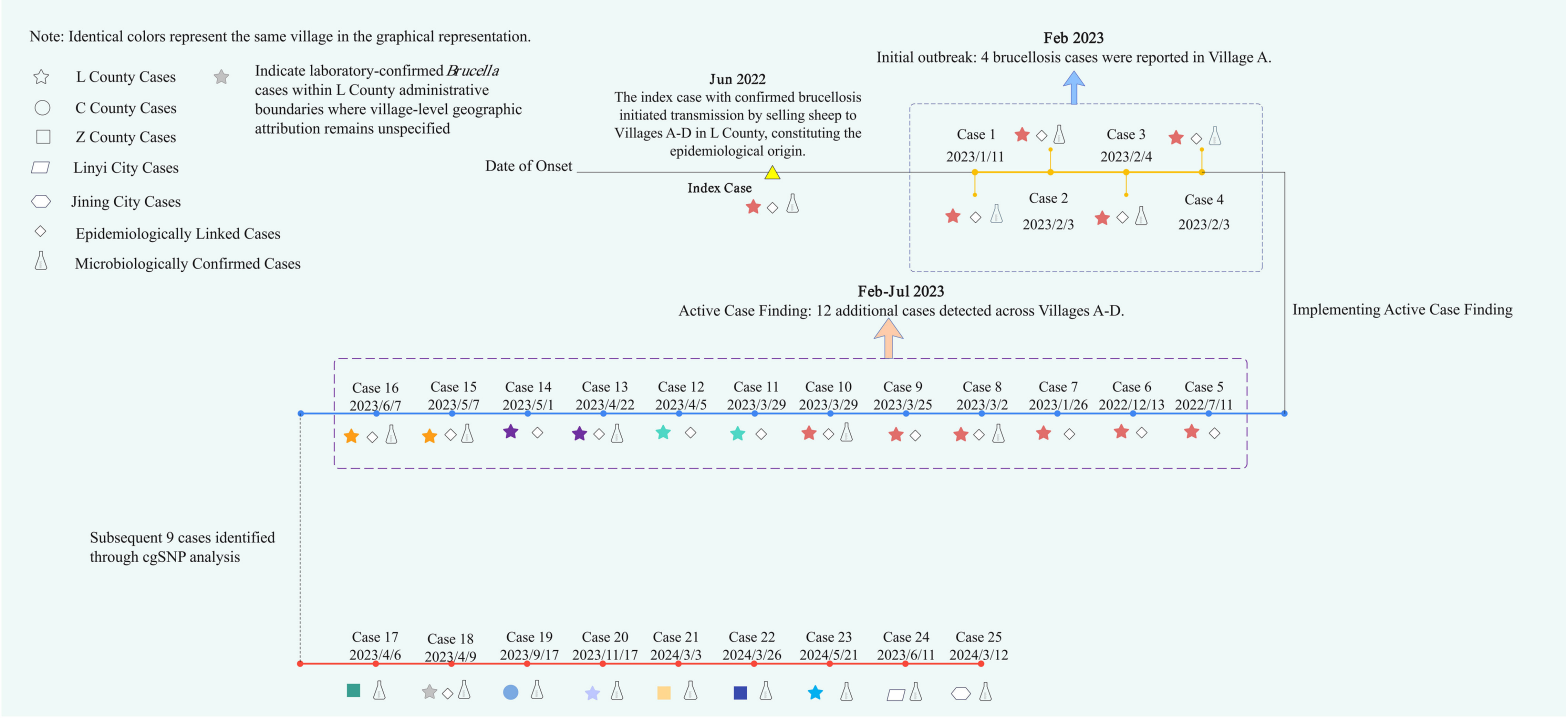
Timeline of the brucellosis outbreak in Weifang City, Shandong Province. Note: Star symbols represent human brucellosis case locations in L County: Red: Village A (n=11); Green: Village B (n=2); Deep Purple: Village C (n=2); Yellow: Village D (n=2); Light Purple: Village E (n=1); Blue: Village F (n=1); Gray: Unspecified location in L County (n=1). Villages A, B, C, D, and F are all within J Town.

These measures identified 12 additional confirmed cases. The cases were distributed as follows: six cases in Village A (Cases 5-10), two each in Villages B (Cases 11-12), C (Cases 13-14), and D (Cases 15-16) (**Figure 2**). In total, the outbreak comprised 16 laboratory-confirmed cases (excluding the index case). All cases had a documented history of purchasing sheep from the index case and unprotected contact with the animals and resided in villages where human-animal cohabitation was common.

### 3.2 Laboratory testing and molecular typing results

#### 3.2.1 Strain isolation and identification

*Brucella* strains were isolated from all 16 outbreak cases and from a historical sample of the index case, yielding ten strains (nine newly cultured and one historical strain) for analysis. All were confirmed as *Brucella* spp. by BCSP31-PCR. Subsequent AMOS-PCR and serum agglutination tests identified nine strains as *B. melitensis* bv. 3 and one strain (from Case 4) as bv. 1.

#### 3.2.2 Genetic typing and tracing based on MLVA

**Figure 3:**
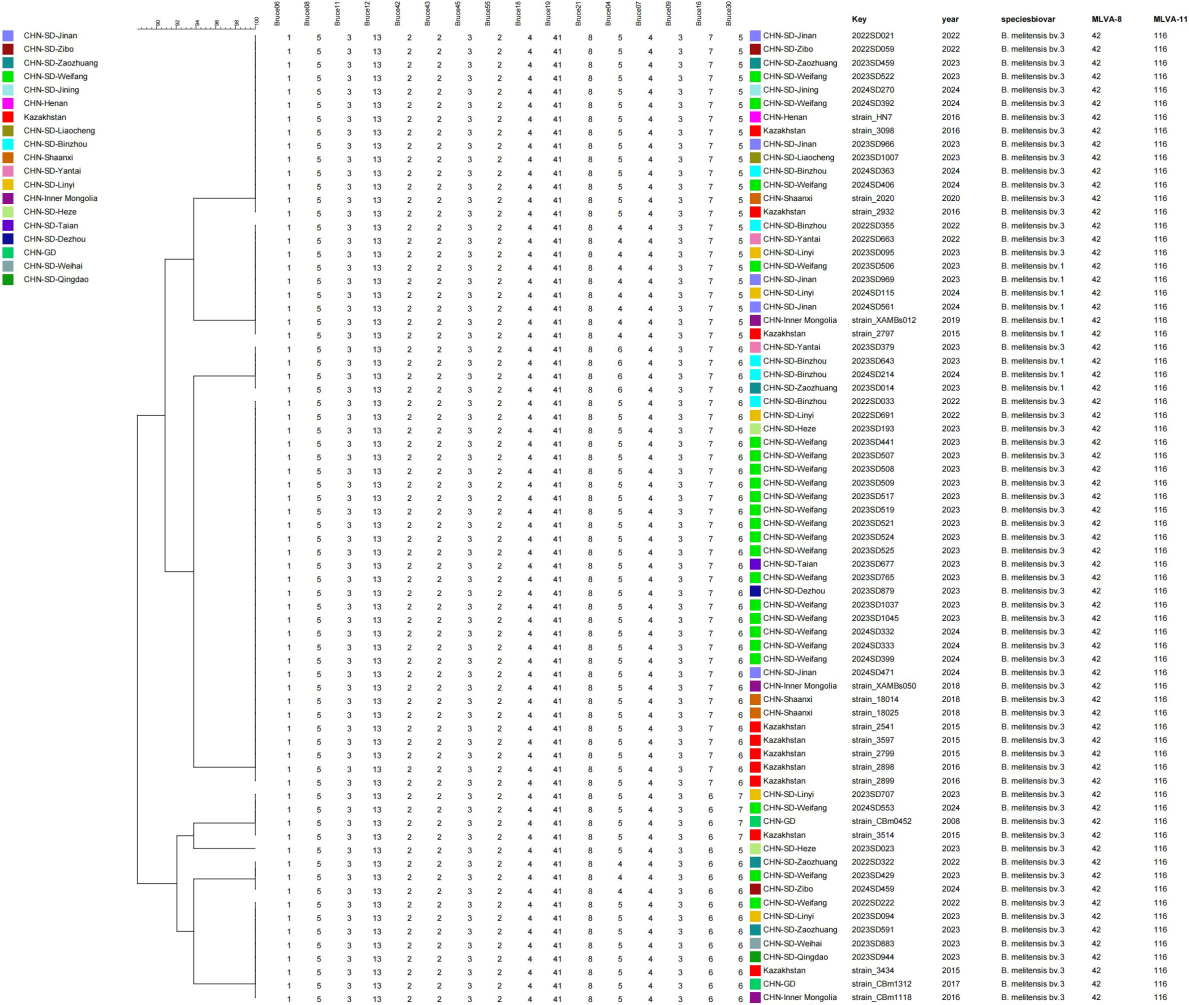
UPGMA dendrogram of *Brucella* isolates based on MLVA-16. Outbreak isolates were analysed together with reference strains from MLVAbank and the Shandong provincial surveillance collection 2022-2024. The scale bar indicates genetic distance; colors denote strain origin.

MLVA-16 typing revealed that all ten strains shared identical MLVA-8 and MLVA-11 profiles, specifically genotypes 42 and 116, respectively. At the MLVA-16 level, eight of the nine bv. 3 outbreak strains (from cases 1-3, 8, 10, 13, 15, 16) exhibited an identical genotype (**Figure 3**). The historical bv. 3 strain from the index case (2022SD222) was highly similar, differing only at locus Bruce16. In contrast, the bv. 1 strain from Case 4 (2023SD506) showed a distinct profile, differing from the bv. 3 cluster at loci Bruce04 and Bruce30.

The predominant bv. 3 MLVA-16 genotype matched 13 historical strains from Shandong Province collected between 2022 and 2024, including 7 from Weifang City and 6 from other cities. Query of a global MLVA database revealed matching genotypes in strains from Shaanxi Province (n=2) and the Inner Mongolia Autonomous Region (n=1) in China, as well as from Kazakhstan (n=5).

#### 3.2.3 cgSNP phylogenetic analysis

**Figure 4:**
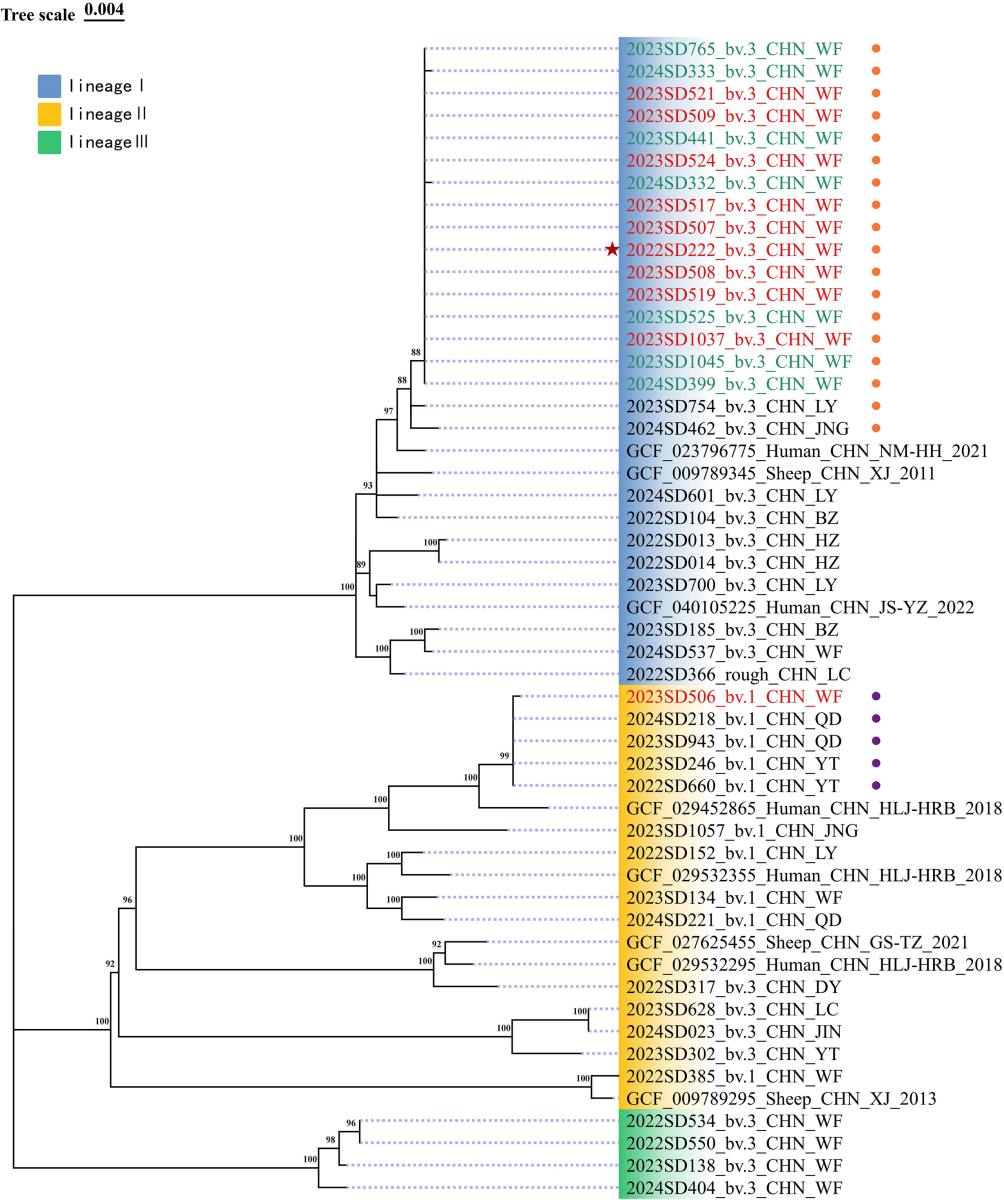
ML phylogenetic tree of *Brucella* strains based on cgSNP analysis. The tree was constructed using isolates from this outbreak, surveillance strains from Shandong Province (2022-2024), and representative Chinese strains from the NCBI database. Strains are differentiated by symbols and colors: the asterisk indicates the index case of this outbreak; red labels denote strains successfully isolated during this outbreak; green labels represent strains isolated from Weifang City that exhibited ≤1 SNP difference from the outbreak *B. melitensis* bv. 3 strains; orange circles encompass all strains with ≤7 SNP differences from the *B. melitensis* bv. 3 outbreak strains; purple circles encompass strains with ≤7 SNP differences from the *B. melitensis* bv. 1 outbreak strain. Major lineages (I, II, III) are indicated.

Phylogenetic analysis based on cgSNP revealed three major lineages (designated I, II, and III). The phylogenetic tree with these lineages is shown in **Figure 4**. The dataset included the outbreak strains, surveillance strains from Shandong Province, and closely related representative domestic strains from public databases. Following a previously established threshold, strains differing by ≤7 SNPs were considered potentially epidemiologically linked. The analysis placed the core transmission cluster of the outbreak within Lineage I. All nine bv. 3 outbreak strains shared an identical cgSNP profile (0 SNP differences) with seven historical strains from Weifang. Two additional historical strains from Shandong (Linyi: n=1; Jining: n=1) were closely related to this cluster, falling within the ≤7 SNPs linkage threshold. This predominant cluster was genetically closest to a human-derived strain from Inner Mongolia (GCF_023796775, 8 SNP differences) and an ovine strain from Xinjiang (GCF_009789345, 15 SNP differences).

The bv. 1 strain from Case 4 (2023SD506) clustered within Lineage II, forming a distinct group with four strains from Shandong (Yantai: n=2; Qingdao: n=2; pairwise SNP differences ≤1). It was genetically closest to a human-derived strain from Heilongjiang Province (GCF_029452865), with 16 SNP differences.

### 3.3 Integrated analysis of case characteristics and molecular epidemiology

#### 3.3.1 Overall characteristics of the cases

A total of 26 outbreak-related cases, including the index case, were analysed by integrating epidemiological and molecular data. Demographic characteristics are shown in **Table 1**. The cohort comprised 14 (53.8%) male and 12 (46.2%) female patients, with a median age of 62 years (range: 8-74). Cases were identified primarily between May 2022 and May 2024, with a distinct epidemic peak between January and June 2023 accounting for 17 cases (65.4%) and a secondary peak of 3 cases in March 2024 (**Figure 1a**). Seasonally, 21 cases (80.8%) were identified in winter and spring (December-May), and 5 (19.2%) in summer and autumn (June-November). The median diagnostic delay was 14.5 days (range: 1-251) (**Figure 1b**). Four cases (15.4%) had prolonged delays, the longest being 251 days.

Geographically, L County was the core epidemic area with 20 cases (76.9%); one case was a resident of Weihai City (Case 18) who had a confirmed exposure history in L County and shared an identical cgSNP genotype (0 SNP difference; **Table 1**) with the main outbreak cluster (**Figure 1c**). Within L County, Village A had the highest burden (11 cases, 42.3%), followed by Villages B-D (2 cases each, 7.7%) and Villages E-F (1 case each, 3.8%). Cross-regional spread involved 6 cases (23.1%): 3 in Z County (11.5%), 1 in C County (3.8%), and 1 each in Linyi and Jining Cities (3.8% each). Overall, Weifang City accounted for 92.3% (24/26) of all cases.

#### 3.3.2 Case association analysis based on molecular and epidemiological evidence

Based on the availability of bacterial strains, molecular typing data, and epidemiological evidence, the 26 cases were classified into four distinct groups (**Table 1**): Group 1 (Molecular and epidemiological evidence supporting linkage, n=10, 38.5%) included the index case and Cases 1-3, 8, 10, 13, 15, 16, and 18. All cases in this group had a clear epidemiological link to the index case’s infected lambs. Their corresponding *B. melitensis* bv. 3 strains were genetically identical, forming a single cluster with 0 SNP differences. Group 2 (Molecular evidence of an independent source, n=1, 3.8%) consisted of Case 4. The *B. melitensis* bv. 1 strain from this case differed from the outbreak’s predominant clone in biotype, MLVA-16 profile, and cgSNP lineage, indicating a separate event. Group 3 (Epidemiological linkage only, n=7, 26.9%) comprised Cases 5-7, 9, 11, 12, and 14. These cases reported relevant exposure histories, but no bacterial strains were obtained for molecular confirmation. Group 4 (Molecular linkage only, n=8, 30.8%) included Cases 17 and 19-25. For these cases, no direct epidemiological link to the index case was established. However, their *B. melitensis* bv. 3 strains were closely related to the main outbreak cluster (≤7 SNP differences).

## 4 Discussion

By integrating field epidemiology with high-resolution genomic techniques, this study reconstructed the transmission chain of a large 2023 brucellosis outbreak in Shandong Province. The outbreak was primarily driven by the local trade of infected sheep, consistent with established knowledge that brucellosis is mainly propagated through direct animal contact (Wang et al., 2020). Notably, molecular typing revealed that the outbreak occurred against a complex endemic background characterized by multiple co-circulating genetic lineages. This finding indicates that in highly endemic areas, while an outbreak may originate from a single introduction, sustained local transmission can be supported by several independent lineages. Such complexity poses a significant challenge to the design and implementation of targeted control measures (Cao et al., 2018; An et al., 2024).

The co-circulation of multiple lineages observed in this outbreak aligns with the national epidemiological pattern of brucellosis in China, where *B. melitensis* is the dominant cause of human disease. Among the 12 known *Brucella* species, *B. melitensis*, *B. abortus*, and *B. suis* are the main human pathogens, with *B. melitensis* being the most virulent and considered the most pathogenic *Brucella* species in humans. In China, human infections caused by *B. abortus* are sporadic and account for a very low proportion of reported cases, much lower than those caused by *B. melitensis* (Huangfu et al., 2025), consistent with its lower pathogenicity (Kneipp et al., 2019). This biological advantage of *B. melitensis*, compounded by the vast sheep and goat population and intensive cross-provincial livestock trade as exemplified by this outbreak, has facilitated its widespread dissemination. At the national level, occupational exposure to infected livestock remains the predominant transmission route. In addition, consumption of raw milk without boiling or pasteurization is a common cultural habit in some Chinese regions and persists among urban residents through informal sales channels, contributing to sporadic brucellosis cases and posing an ongoing public health risk.

This study demonstrates that high-resolution molecular typing based on WGS uniquely overcomes the spatiotemporal constraints of traditional epidemiology (van der Werf and Ködmön, 2019; Sundermann et al., 2022). While MLVA-16 can rapidly identify major transmission clusters, its limited resolution may lead to misinterpretation (Tan et al., 2023). Nevertheless, its standardized 16-locus panel remains valuable for rapid preliminary screening and direct comparison with publicly available databases for global strain comparison. For instance, the variation observed at the Bruce16 locus between the index case and outbreak strains was resolved by cgSNP analysis, which confirmed their clonal origin (0 SNP differences), suggesting microevolution within a single chain. cgSNP analysis further revealed transmission details missed by conventional methods. First, the outbreak cluster was closely related (≤6 SNP differences) to strains from Linyi and Jining within Shandong, indicating an unrecognized inter-city transmission network. Second, its genetic similarity (≤15 SNP differences) to strains from northern pastoral regions (Inner Mongolia, Xinjiang) provides molecular evidence that inter-provincial animal movement contributed to local spread (Jackson et al., 2016). This aligns with findings from Sichuan, where the dominant local genotype was also shared across northern provinces, highlighting the role of cross-regional livestock trade in *Brucella* dissemination (Li et al., 2025). Case 18 (a Weihai resident infected in L County) exemplifies how reliance on residential data alone can obscure true transmission routes, whereas molecular tracing captures population movement across administrative boundaries. The integrated approach was validated, with 38.5% (10/26) of cases showing complete concordance between molecular and epidemiological evidence, thereby corroborating the core transmission chains. Group 3 and Group 4 cases, together accounting for more than half (57.7%, 15/26) of the outbreak, highlight two complementary gaps in current brucellosis surveillance. Group 3 cases, lacking bacterial isolates, reflect the challenge of timely specimen collection in endemic areas, a key bottleneck for genomic surveillance. Group 4 cases, identified only through molecular typing, reveal transmission networks that extend beyond epidemiological records, likely driven by unregulated livestock trade. These findings demonstrate that neither epidemiological investigation nor WGS alone is sufficient; integrating both is essential to resolve outbreaks in high-endemic settings.

Phylogenetic analysis revealed the co-circulation of multiple *Brucella* lineages in the local population. Besides the outbreak-dominant Lineage I, two independent lineages were identified. Lineage III consists of closely related local bv. 3 strains that are genetically distinct (>50 SNP differences) from the outbreak cluster, indicating a separate, endemic transmission chain. Lineage II includes the bv. 1 strain from Case 4, which clusters closely (≤1 SNP difference) with strains from Yantai and Qingdao, suggesting an independent introduction or another localized transmission chain. The source of infection for Case 4 was not identified through conventional epidemiology, and its molecular profile clearly differed from the outbreak cluster, further demonstrating the limitation of traditional approaches in detecting multiple concurrent transmission sources in high-endemic settings.

The epidemiological findings of this study have direct implications for disease control. The median diagnostic delay was 14.5 days, exceeding the previously reported national median, with one case delayed by 251 days (Li et al., 2020). This gap was largely attributable to non-specific clinical presentations, limited awareness among primary care physicians, and low health-seeking behavior among patients (Al Dahouk et al., 2007; Cama et al., 2019). In the context of this poverty-associated disease, diagnostic delays can initiate a detrimental cycle. Delayed diagnosis leads to chronic, complicated illness that requires more expensive treatment. For livestock-dependent households, this may result in substantial medical costs and income loss, potentially exacerbating poverty. This may further deter timely care-seeking (Di Bari et al., 2022). Prolonged delays increase the risk of chronic infection and may facilitate further transmission if patients remain undiagnosed. To reduce diagnostic delays, public health policies should prioritize systematic training programs for primary care physicians to improve their ability to recognize and diagnose brucellosis. Such training should be integrated into routine professional development with periodic assessments. Equipping township health centers with rapid diagnostic tools and establishing direct reporting and consultation mechanisms with local CDCs would further facilitate early diagnosis in high-endemic areas. Case incidence peaked between March and June 2023, with another rise in March 2024, reflecting the known high-incidence season (March-August) in Shandong Province (Yu et al., 2023). This seasonal pattern aligns with periods of increased human-animal contact during key farming activities such as lambing and shearing. These observations highlight a critical window for intervention by reinforcing quarantine, occupational protection, and public education before the high-risk season to effectively reduce transmission.

Based on this integrated analysis, we propose three key recommendations to enhance brucellosis surveillance and control: (1) Strengthen the molecular surveillance network. Implement continuous monitoring based on WGS in key agricultural and pastoral regions. This approach will supplement lower resolution genotyping and allow for real-time detection of newly introduced lineages and locally circulating clones. (2) Develop an integrated, cross-sectoral data platform. A unified platform should bridge the gap between animal and human health sectors by consolidating animal quarantine records, logs of livestock movement across regions, and detailed human case exposure histories. Such integration is essential for robust linkage and validation in molecular epidemiology. (3) Enhance frontline capacity and targeted interventions. Optimize protocols for the collection and submission of samples from the acute phase of illness to improve strain isolation rates. Increase diagnostic training for primary healthcare providers and deliver focused health education to high-risk occupational groups (e.g., livestock farmers, slaughterhouse workers) regarding specific exposure risks and the importance of seeking early medical consultation.

Several limitations should be acknowledged. This study analyzed only human-derived *Brucella* isolates; animal and environmental samples were not available. Consequently, direct molecular evidence linking human cases to infected animals or contaminated environments could not be obtained. Additionally, inherent limitations of field investigations (e.g., recall bias, potential underreporting) mean that no case finding approach can be entirely gap-free, though our multi-faceted strategy helped mitigate such limitations. The eight Group 4 cases, identified by molecular typing of archived isolates, lacked detailed exposure data (e.g., contact with the index case’s flock, exposure time/place, transmission route). This hindered definitive epidemiological linkage despite ≤7 SNP differences to the outbreak cluster, limiting resolution of transmission pathways. For the seven epidemiologically linked Group 3 cases, no *Brucella* strains were isolated, primarily due to delayed blood collection or prior antibiotic use. To improve isolation success, blood samples should be collected during the acute febrile phase and ideally before antibiotic administration. On-site training for primary care clinicians on timely specimen collection and transport procedures should also be routinely implemented to increase the isolation rate and strengthen genomic surveillance capacity. Importantly, this did not affect the core conclusions regarding the outbreak’s origin and spread, which were robustly supported by the available isolate data and clear epidemiological links. Future prospective, multicenter studies incorporating broader animal and environmental sampling are needed to fully elucidate regional transmission dynamics.

## 5 Conclusion

This study integrated epidemiological investigation with molecular tracing to characterize the transmission chain, pathogen profile, and cross-regional links of a brucellosis outbreak in Weifang City, Shandong Province. The outbreak originated from the local trade of infected animals and spread through an informal trading network via a multilevel diffusion pattern. High-resolution cgSNP analysis revealed micro-evolution within the core strain cluster, genetic connections to strains from other pastoral areas within Shandong, and the co-existence of locally circulating lineages alongside introduced cases. These findings highlight the essential role of molecular tracing in overcoming the limitations of traditional epidemiology, uncovering hidden transmission chains, and evaluating cross-regional risks. Strengthening source control is crucial for controlling the spread of brucellosis and other zoonotic diseases. This must be accompanied by advancing technical capacity and fostering multisectoral collaboration to establish an integrated monitoring, alert, and response system.

## Data Availability

The datasets presented in this study can be found in online repositories. The names of the repository/repositories and accession number(s) can be found in the article/**Supplementary Table 2**.
