# Correction: Integrating epidemiology with genomic tracing to uncover concealed transmission networks in a brucellosis outbreak, Shandong, China

**DOI:** 10.3389/fcimb.2026.1868203

**Published:** 2026-05-11

**Authors:** Chanjuan Huangfu, Min Wang, Junjie Fan, Xiujun Ma, Haizong Zhang, Jing Yuan, Kuo Han, Ti Liu, ShuJun Ding, Zengqiang Kou, Yan Li

**Affiliations:** 1 School of Public Health and Health Management, Shandong First Medical University, Jinan, China; 2 Clinical Laboratory, Children’s Hospital Affiliated to Shandong University, Jinan Children’s Hospital, Jinan, China; 3 Department of Infectious Disease Prevention and Control, Weifang Center for Disease Control and Prevention, Weifang, China; 4 Shandong Provincial Key Laboratory of Intelligent Monitoring, Early Warning, Prevention and Control for Infectious Diseases, Shandong Center for Disease Control and Prevention, Jinan, China; 5 Department of Infectious Disease Prevention and Control, Linqu County Center for Disease Control and Prevention, Weifang, China

**Keywords:** *Brucella melitensis*, brucellosis, molecular epidemiology, molecular typing, outbreaks, whole-genome sequencing

In the published article, the equal contribution annotations for the first three authors were inadvertently omitted. The authors Chanjuan Huangfu, Min Wang, and Junjie Fan should be marked with asterisks (†) as joint first authors. The correct author list with annotations reads:

“Chanjuan Huangfu^1†^, Min Wang^2†^, Junjie Fan^3†^, Xiujun Ma ^4^,Haizong Zhang^5^, Jing Yuan^5^, Kuo Han^4^, Ti Liu^4^, ShuJun Ding^4^, Zengqiang Kou^1,4^* and Yan Li^4^*”

The original version of this article has been updated.

