# Supplementary material for: Integrating epidemiology with genomic tracing to uncover concealed transmission networks in a brucellosis outbreak, Shandong, China
Source: Front Cell Infect Microbiol. 2026 Apr 21;16:1794629. doi: 10.3389/fcimb.2026.1794629 (PMC13138912; doi:10.3389/fcimb.2026.1794629)
Supplement: Supplementary file 1 [file Table1.docx]

**Brucellosis Case Investigation Form**

Case ID □□□□□

Province ______ City ______ County/District ______ Township/Subdistrict ______

**1. Demographic Information**

1.1 Patient Name: ______ Contact Number: ______

1.2 Sex: ①Male ②Female

1.3 Age: ______ years

1.4 Ethnicity: ______

1.5 Occupation: ①Farmer (livestock breeding, trading, slaughtering, raw meat sales, fur/leather processing, animal product transport) ②Worker (livestock breeding, trading, slaughtering, raw meat sales, fur/leather processing, animal product transport) ③Veterinarian ④Medical staff ⑤Student ⑥Public servant ⑦Teacher ⑧Other: ______

1.6 Address of Onset: ______ County/District ______ Township ______ Village/Street No. ______

1.7 Home Address: ______ County/District ______ Township ______ Village/Street No. ______

1.8 Date of Onset (YYYY/MM/DD): ______

1.9 Date of Hospitalization (YYYY/MM/DD): ______

1.10 Date of Reporting (YYYY/MM/DD): _____

1.11 Hospital Name: ______

**2. Clinical Manifestations**

2.1 Symptoms and Signs

2.1.1 Fever: ①Yes ②No

  If yes: Duration ______ days, Maximum temperature ______℃

2.1.2 Hyperhidrosis: ①Yes ②No

2.1.3 Muscle/Joint Pain: ①Yes ②No

2.1.4 Fatigue: ①Yes ②No

2.1.5 Hepatomegaly: ①Yes ②No

2.1.6 Splenomegaly: ①Yes ②No

2.1.7 Lymphadenopathy: ①Yes ②No

2.1.8 Orchitis: ①Yes ②No

2.2 Laboratory Tests

2.2.1 Rose Bengal Plate Test (RBPT): ①Positive ②Negative

2.2.2 Standard Tube Agglutination Test (SAT) titer ≥1:100: ①Yes ②No

2.2.3 Pathogen Isolated: ①Yes ②No

2.2.4 If yes, specimen type: ①Blood ②Bone marrow ③Other body fluids ④Excreta

2.3 Clinical Diagnosis: ______

2.4 Treatment

2.4.1 Antibiotics: ①Yes ②No

2.4.2 Vaccine therapy: ①Yes ②No

2.4.3 Traditional Chinese medicine: ①Yes ②No

2.4.4 Other: ①Yes (specify: ______) ②No

2.5 Outcome: ①Recovered ②Improved ③Uncured ④Deceased: (YYYY/MM/DD) ______

**3. Epidemiological Investigation**

3.1 Exposure History (Within 3 Weeks Before Onset)

3.1.1 Animal contact: ①Yes ②No

   If yes, animal species (multiple allowed): ①Sheep/goat ②Cattle ③Pig ④Mink ⑤Fox ⑥Other: ______

3.1.2 Grazing/feeding: ①Yes ②No

3.1.3 Slaughtering or meat/organ processing: ①Yes ②No

3.1.4 Shearing wool or fur/leather processing/transport: ①Yes ②No

3.1.5 Assisting parturition or handling aborted fetuses: ①Yes ②No

3.1.6 Breeding assistance: ①Yes ②No

3.1.7 Animal disease investigation (veterinarian): ①Yes ②No

3.1.8 Contact with urine/feces of infected animals: ①Yes ②No

3.1.9 Consumption of raw milk: ①Yes ②No

3.1.10 Hunting or consuming wild animals: ①Yes ②No

3.1.11 Presence of wounds during animal contact: ①Yes ②No

3.1.12 Household member with brucellosis: ①Yes (date of onset: YYYY/MM/DD): _____ ②No

**3.2 Personal Protective Measures**

3.2.1 Use of protective measures during animal contact: ①Yes ②No

   If yes (multiple allowed): ①Gloves ②Protective clothing ③Mask ④Other:______

3.2.2 Handwashing after animal contact: ①Yes ②No

   If yes, commonly used (multiple allowed): ①Water ②Soap ③Disinfectant ④Other: ______

3.2.3 Regular laundry: ①Yes ②No

   If yes, frequency: ______ times/week

3.2.4 Livestock raising: ①Yes ②No

  3.2.4.1 Species and quantity (multiple allowed): ①Sheep/goat ______ heads ②Cattle ______ heads ③Pig ______ heads ④Other ______, ______ heads

  3.2.4.2 Free-range in courtyard: ①Yes ②No

  3.2.4.3 Cleaning/disinfection of housing, pens, equipment: ①Yes ②No

    If yes, frequency: ______ times/month

  3.2.4.4 Manure treatment (burning, burying): ①Yes ②No

3.3 Consumption of mutton/beef within 3 weeks before onset: ①Yes ②No

3.3.1 Cooking method (multiple allowed): ①Hot pot ②Stewed ③Grilled ④Other: ______

 3.3.2 Description of processing and consumption (e.g., shape of grilled meat skewers): ______

3.4 Past medical history: ______

3.5 Date of confirmation (YYYY/MM/DD): ______

3.6 Suspected source of infection, transmission route, and vehicle: ______

**4. Investigation Summary**______________________________

Investigator: ______ Date of investigation (YYYY/MM/DD): ______
